# Autoimmune Manifestations Following COVID-19 Infection: A Systematic Review

**DOI:** 10.7759/cureus.112421

**Published:** 2026-07-10

**Authors:** Sara Omer Mohamed Omer, Riem Eltahir, Shaza Bakri Osman Elsayed, Mahmoud Abdelghany Eldyasty Ahmed Eid, Rihab Abdelhafiz Hassan Khattab, Eltayeb Osman Elfaki Omer, Mohamed Kamal Gomaa Abdelfattah, Azhar Samir Mustafa Alsalhi, Noran Fatihelrahman Elawad Ahmed

**Affiliations:** 1 General Internal Medicine, Al Dhafra Hospitals, Abu Dhabi Health Service Company (SEHA), Abu Dhabi, ARE; 2 Neonatology, Heartlands Hospital, University Hospitals Birmingham NHS Foundation Trust, Birmingham, GBR; 3 Rheumatology, Croydon Health Services NHS Trust, Croydon, GBR; 4 Dermatology, Alazhar University of Cairo, Cairo, EGY; 5 Pathology, Khartoum University, Khartoum, SDN; 6 Endocrinology, Dr. Sulaiman Al Habib Hospital, Riyadh, SAU; 7 Rheumatology, Al-Azhar University, Cairo, EGY; 8 General Medicine, Independent Practice, Amman, JOR; 9 General Medicine, The National Ribat University, Khartoum, SDN

**Keywords:** autoimmune disease, autoimmunity, covid-19, new-onset, sars-cov-2, systematic review

## Abstract

Coronavirus disease 2019 (COVID-19) has been linked to immune dysregulation and autoantibody formation, raising concern that severe acute respiratory syndrome coronavirus 2 (SARS-CoV-2) infection may precipitate new-onset autoimmune disease. This systematic review synthesises controlled observational evidence on the incidence and risk of autoimmune manifestations following confirmed COVID-19.

Following the PRISMA (Preferred Reporting Items for Systematic reviews and Meta-Analyses) 2020 guidelines, electronic databases were searched from inception upto the search date for original cohort or case-control studies comparing incident autoimmune disease in individuals with versus without confirmed COVID-19. Eligibility was structured using the PICOS (Population, Intervention/Exposure, Comparator, Outcome, and Study design) framework. Methodological quality was appraised using the Newcastle-Ottawa Scale and certainty of evidence using the GRADE approach. Findings were synthesised narratively.

Eight large cohort studies, drawn from multiple national health systems and collectively encompassing tens of millions of individuals, met the inclusion criteria. Confirmed SARS-CoV-2 infection was consistently associated with an increased risk of incident autoimmune disease spanning rheumatological, dermatological, vasculitic, endocrine, gastrointestinal, haematological, and neurological domains, with adjusted hazard ratios ranging from approximately 1.3 to 3.0. Connective tissue, vasculitic, and antibody-mediated disorders were most frequently implicated. Several studies demonstrated a severity-dependent gradient and attenuation of risk over time, and one cohort reported a reduced risk among vaccinated individuals. The overall certainty of evidence was low to moderate.

Confirmed COVID-19 is associated with an increased, though in absolute terms modest, risk of new-onset autoimmune disease, most reproducibly affecting connective tissue, vasculitic, and antibody-mediated conditions. The observational evidence supports clinical vigilance, particularly after severe infection, while prospective studies with validated outcomes and infection comparators are needed to confirm causality.

## Introduction and background

Coronavirus disease 2019 (COVID-19), caused by severe acute respiratory syndrome coronavirus 2 (SARS-CoV-2), was first characterised as a predominantly respiratory illness, with fever, cough, fatigue, myalgia, and dyspnoea dominating the early clinical descriptions from Wuhan, China [1]. It rapidly became clear, however, that SARS-CoV-2 provokes a far broader host response than respiratory epithelial injury alone. Severe infection is accompanied by exaggerated and misdirected innate and adaptive immune activation, and high-throughput screening of infected individuals has demonstrated that patients with COVID-19 develop a strikingly diverse repertoire of functional autoantibodies, including reactivities against cytokines, chemokines, complement components, and cell-surface and tissue antigens [2]. These observations positioned COVID-19 not merely as an infection but as a potential trigger of durable immune dysregulation, raising the question of whether SARS-CoV-2 might precipitate frank autoimmune disease in susceptible hosts.

The notion that a viral infection can initiate autoimmunity is well-established. Loss of immunological self-tolerance after infection is classically attributed to mechanisms such as molecular mimicry, bystander activation, and epitope spreading, and bioinformatic and experimental work has identified substantial sequence and conformational homology between SARS-CoV-2 proteins and human self-antigens that could drive cross-reactive immune responses [3]. Consistent with this, immune-mediated complications were reported from the earliest months of the pandemic; Guillain-Barré syndrome, a prototypical post-infectious autoimmune neuropathy, was described in temporal association with SARS-CoV-2 infection in northern Italy in 2020 [4]. Autoimmunity has since been proposed as one of the principal mechanistic hypotheses underlying the heterogeneous, multi-organ presentation of post-acute sequelae of SARS-CoV-2 infection, or long COVID [5]. Beyond case-level descriptions, dataset-level signals accumulated for specific conditions, including an increased incidence of rheumatoid arthritis after COVID-19 [6] and a higher risk of new-onset type 1 diabetes among children following SARS-CoV-2 infection compared with other respiratory infections [7].

Despite this rapidly expanding literature, much of the early evidence consisted of isolated case reports and small series that could neither establish incidence nor distinguish a genuine association from coincidental co-occurrence during a global pandemic. The subsequent emergence of large, population-based and matched cohort studies--drawing on national health-insurance systems and multinational electronic health-record networks--now permits a more rigorous appraisal of whether, and to what degree, SARS-CoV-2 infection is associated with new-onset autoimmune disease. A systematic synthesis of these controlled, population-level studies is therefore warranted to consolidate the direction and magnitude of risk, to characterise which conditions are most consistently implicated, and to identify the methodological limitations that temper causal interpretation. Accordingly, this systematic review was conducted to summarise the evidence from controlled observational studies examining the incidence and risk of autoimmune manifestations following confirmed COVID-19 infection.

## Review

Methods

Study Design

This systematic review was conducted and reported in accordance with the Preferred Reporting Items for Systematic Reviews and Meta-Analyses (PRISMA) 2020 statement [8].

Eligibility Criteria

Studies were eligible if they were original, peer-reviewed, controlled observational studies (cohort or case-control designs) reporting the incidence or risk of new-onset (incident) autoimmune or autoinflammatory disease following laboratory- or diagnosis-confirmed COVID-19, relative to a comparator group without COVID-19. Narrative and systematic reviews, meta-analyses, editorials, commentaries, conference abstracts, preprints, case reports, and case series without a comparator were excluded, as were studies reporting only vaccination-associated outcomes, flares of pre-existing autoimmune disease, or acute multisystem inflammatory syndrome without incident autoimmune diagnoses. The full eligibility framework, structured according to the Population, Intervention/Exposure, Comparator, Outcome, and Study design (PICOS) schema [9], is presented in Table 1.

**Table 1:** Eligibility criteria according to the PICOS framework

PICOS element	Inclusion criteria	Exclusion criteria
Population	Individuals of any age with confirmed COVID-19	Populations without confirmed SARS-CoV-2 infection
Exposure	Confirmed SARS-CoV-2 infection (PCR, antigen, or coded diagnosis)	Vaccination as the sole exposure; suspected or unconfirmed infection
Comparator	Contemporaneous or matched individuals without COVID-19 (± other infection comparators)	No comparator group
Outcome	Incident (new-onset) autoimmune or autoinflammatory disease; incidence or relative risk	Flares of pre-existing disease; autoantibodies without clinical disease; mortality only
Study design	Original cohort or case–control studies	Reviews, meta-analyses, editorials, conference abstracts, preprints, case reports or series
Other	Peer-reviewed; English language; human studies	Non–peer-reviewed; non-human; full text unavailable

Information Sources and Search Strategy

A systematic search of MEDLINE/PubMed, Embase, Web of Science, and the Cochrane Library was performed from database inception to May, 2026. The search combined controlled vocabulary and free-text terms for the exposure (e.g., “COVID-19,” “SARS-CoV-2”) and the outcome (e.g., “autoimmune disease,” “autoinflammatory,” “connective tissue disease,” and named conditions such as “rheumatoid arthritis,” “systemic lupus erythematosus,” and “vasculitis”). Reference lists of relevant articles were hand-searched to identify additional eligible studies. The detailed search strings for each database is provided in the Appendices section.

Study Selection and Data Extraction

**Figure 1:**
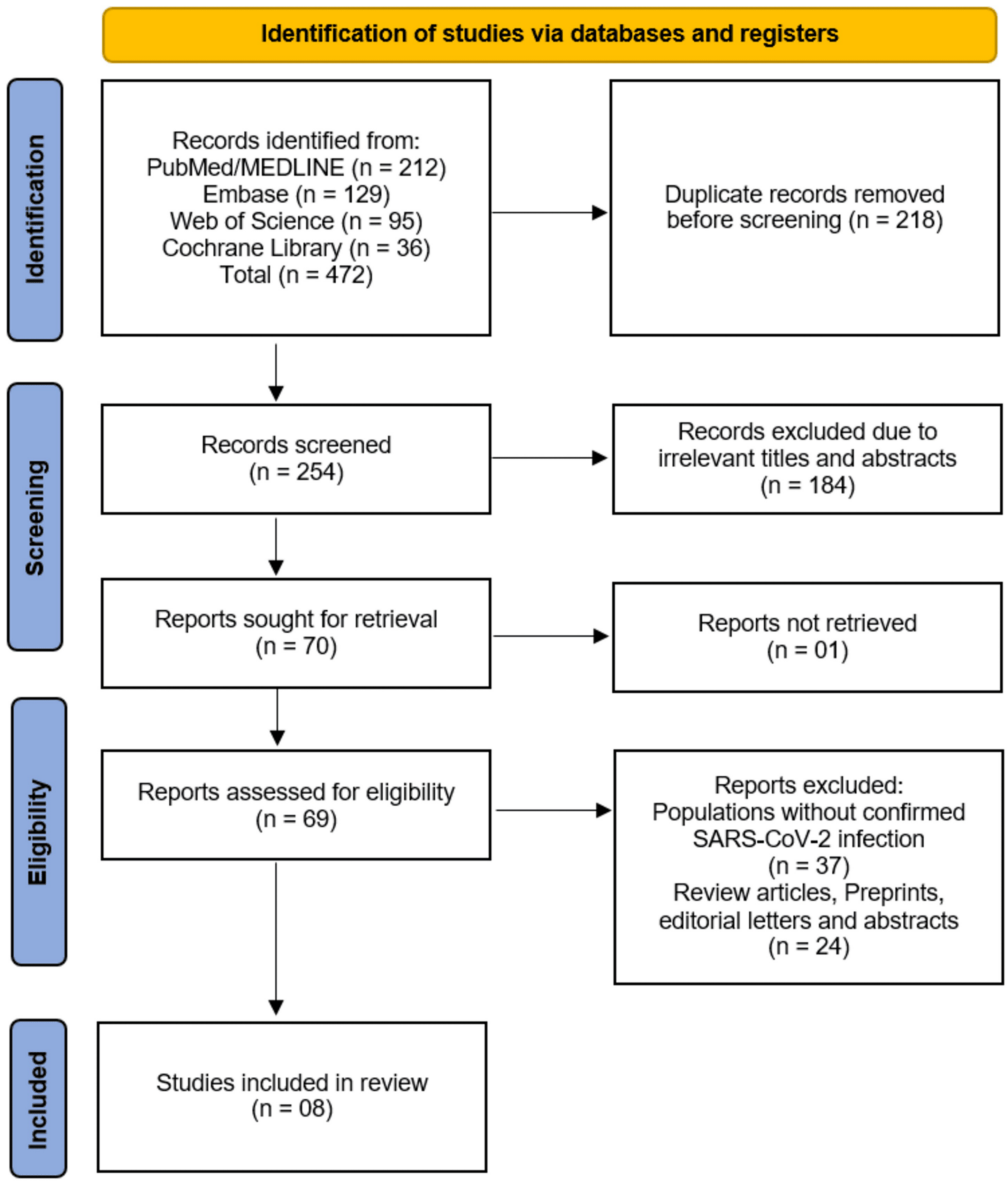
PRISMA flowchart of the study selection process

Following removal of duplicates, two reviewers independently screened titles and abstracts and then assessed full texts against the eligibility criteria, with disagreements resolved by discussion or by a third reviewer. The PRISMA flow of study selection is summarised in Figure 1. From each included study, two reviewers independently extracted the first author, year, country and data source, study design, exposure ascertainment, sample sizes of the exposed and comparator groups, follow-up period, autoimmune outcomes assessed, and the reported effect estimates with 95% confidence intervals (CIs).

Risk-of-Bias and Certainty Assessment

Methodological quality was independently appraised using the Newcastle-Ottawa Scale (NOS) for cohort studies, which evaluates the selection of cohorts, comparability, and ascertainment of outcomes across a maximum of nine stars [10]. The overall certainty of evidence for each principal outcome was rated using the Grading of Recommendations Assessment, Development and Evaluation (GRADE) approach, considering risk of bias, inconsistency, indirectness, imprecision, and publication bias, together with factors that may increase certainty such as a large magnitude of effect and an exposure-response gradient [11]. Given the consistent inclusion of large, population-based datasets and the absence of a quantitative meta-analysis, findings were synthesised narratively.

Results

Study Selection and Characteristics

Eight original controlled cohort studies met the eligibility criteria [12-19]. The studies were geographically diverse, comprising two United States or multinational electronic health-record network analyses [12,17], one German [13], one United Kingdom [14], three South Korean [15,18,19] (one of which was a binational Korea-Japan analysis [18]), and one Hong Kong [16] population-based study. All employed a retrospective cohort design with a non-COVID-19 comparator, and collectively, they encompassed tens of millions of individuals. Exposure was ascertained by polymerase chain reaction, antigen testing, or coded diagnosis, and most studies applied propensity score matching or inverse probability weighting to balance baseline characteristics. The characteristics of the included studies are presented in Table 2.

**Table 2:** Characteristics of the included studies

Study, year, ref	Country / data source	Design	COVID-19 exposed (n)	Comparator (n)	Study period / follow-up
Chang (2023) [12]	USA / TriNetX US Collaborative Network	Retrospective, propensity-matched cohort	888,463	2,926,016	Jan 2020–Dec 2021
Tesch (2023) [13]	Germany / statutory health-insurance claims	Matched cohort (1:3)	PCR-confirmed cases	Matched non-COVID controls	Index to Jun 2021
Syed (2023) [14]	UK / CPRD Aurum primary care	Matched cohort	458,147	1,818,929	2020–2021
Lim (2023) [15]	South Korea / K-COV-N nationwide	Population-based cohort (IPW)	354,527	6,134,940	Oct 2020–Dec 2021
Peng (2023) [16]	Hong Kong / territory-wide records	Retrospective cohort (IPTW)	1,028,721	3,168,467	Apr 2020–Nov 2022
Hileman (2024) [17]	USA / TriNetX global network	Propensity-matched cohort	3,908,592 (total)	3,908,592 (total)	Jan 2020–Mar 2023
Kim (2024) [18]	South Korea + Japan / K-COV-N + JMDC	Binational, propensity-matched cohort	COVID-19 cases	Influenza + uninfected	2020–2021; ≤12 mo
Heo (2024) [19]	South Korea / K-COV-N nationwide	Retrospective cohort (IPW)	3,145,388	3,767,039	Oct 2020–Dec 2022; >180 d

Synthesis of Autoimmune Outcomes

Across the included studies, SARS-CoV-2 infection was associated with an increased risk of incident autoimmune disease, spanning rheumatological, dermatological, endocrine, gastrointestinal, haematological, and neurological systems, although the magnitude and breadth of the association varied considerably with the data source, comparator, and length of follow-up (Table 3). The largest effect sizes were reported in the United States network analysis by Chang et al., in which the COVID-19 cohort showed markedly higher risks of rheumatoid arthritis, ankylosing spondylitis, systemic lupus erythematosus, and several connective tissue diseases [12]. More conservative but consistently positive associations were reported in the territory-wide Hong Kong cohort, where antibody-mediated and dermatological conditions, such as immune thrombocytopenia, antiphospholipid syndrome, pemphigoid, and Graves disease, were prominent [16]. Two studies focused on the dermatological and connective tissue spectrum in Korea and documented severity-dependent increases in alopecia, vitiligo, psoriasis, vasculitis, and inflammatory bowel disease [15,19], while the binational Korea-Japan study demonstrated a positive but time-attenuated association with autoimmune inflammatory rheumatic disease that was reproduced across two independent national datasets and persisted relative to an influenza comparator [18].

**Table 3:** Principal autoimmune outcomes and effect estimates RA: rheumatoid arthritis; AS: ankylosing spondylitis, SLE: systemic lupus erythematosus; SSc: systemic sclerosis; MCTD: mixed connective tissue disease; IBD: inflammatory bowel disease; T1D: type 1 diabetes; IMID: immune‐mediated inflammatory disease; UC: ulcerative colitis; ITP: immune thrombocytopenia; APS: antiphospholipid syndrome; MS: multiple sclerosis; AIRD: autoimmune rheumatic diseases

Study, Ref	Conditions most strongly implicated	Direction	Selected effect estimates (95% CI)
Chang [12]	RA, AS, SLE, Sjögren, SSc, MCTD, vasculitis, psoriasis, IBD, T1D	Increased	RA aHR 2.98 (2.78–3.20); SLE 2.99 (2.68–3.34); AS 3.21 (2.50–4.13); vasculitis 1.96 (1.74–2.20)
Tesch [13]	Broad spectrum incl. arthropathies, vasculitis, autoimmune thyroiditis, psoriasis	Increased	Higher incidence of incident autoimmune disease overall
Syed [14]	Composite IMID; T1D, IBD, psoriasis	Increased	Increased risk of composite IMID outcome
Lim [15]	Alopecia, vitiligo, psoriasis, vasculitis, Crohn, UC, RA, Sjögren, AS	Increased (severity-dependent)	Multiple connective tissue disorders elevated; risk tracked with severity
Peng [16]	ITP, APS, pemphigoid, Graves, MS, RA, spondyloarthritis, vasculitis	Increased	MS aHR 2.66 (1.17–6.05); pemphigoid 2.39 (1.83–3.11); APS 2.12 (1.47–3.05); ITP 2.10 (1.82–2.43)
Hileman [17]	Cutaneous vasculitis, polyarteritis nodosa, hypersensitivity angiitis	Increased (8/24 ADs)	Cutaneous vasculitis aHR 1.82 (1.55–2.13); PAN 1.76 (1.15–2.70)
Kim [18]	Composite AIRD; inflammatory arthritis, connective tissue disease	Increased (time-attenuated)	Elevated AIRD risk vs uninfected and vs influenza; greater with severe COVID-19
Heo [19]	RA, SLE, Crohn, alopecia, vasculitis	Increased (long-term)	Elevated long-term risk; greater for severe, Delta-period, and unvaccinated cases

Three cross-cutting patterns emerged from the synthesis. First, the direction of association was consistent: every included study reported an overall increase in the risk of one or more incident autoimmune conditions after COVID-19, with vasculitic, connective tissue, and antibody-mediated disorders among the most frequently implicated [12,15-17,19]. Second, several studies identified an exposure-response relationship, in which more severe acute COVID-19 conferred a higher subsequent risk of autoimmune disease [15,18,19]. Third, the available data suggested temporal and protective modifiers: the binational study showed the attenuation of risk over the 12 months of follow-up [18], whereas the Hong Kong cohort reported that completion of COVID-19 vaccination was associated with a reduced risk of several infection-associated autoimmune conditions [16]. Notably, the magnitude of association differed by an order of magnitude across studies, from hazard ratios near 1.3 to nearly 3.0 for comparable conditions, underscoring substantial heterogeneity attributable to differing case definitions, comparator selection, surveillance intensity, and circulating variants.

Certainty of Evidence (GRADE)

Because all included studies were non-randomised, the body of evidence began at low certainty under the Grading of Recommendations, Assessment, Development, and Evaluation (GRADE) framework. Certainty was supported by the large magnitude of effect and the exposure-response gradient observed for several outcomes, but was offset by between-study inconsistency, the potential for surveillance and detection bias inherent in electronic health record data, and residual confounding. The resulting certainty ratings ranged from low to moderate across outcome domains (Table 4).

**Table 4:** GRADE certainty of evidence assessment GRADE: Grading of Recommendations, Assessment, Development, and Evaluation; ITP: inflammatory bowel disease; APS: antiphospholipid syndrome; T1D: type 1 diabetes; IBD: inflammatory bowel disease

Outcome domain	Studies	Design	Key downgrading factors	Upgrading factors	Certainty
Any incident of autoimmune disease	[12–19]	Observational	Inconsistency; detection bias	Large effect; exposure–response	Moderate
Connective tissue / rheumatic disease	[12,15,18,19]	Observational	Residual confounding	Large effect	Low
Vasculitis	[12,16,17]	Observational	Imprecision (rare outcome)	Consistency of direction	Low
Antibody-mediated disorders (ITP, APS)	[16]	Observational	Single study; imprecision	Large effect	Low
Endocrine/gastrointestinal (T1D, IBD)	[12,14]	Observational	Indirectness; confounding	—	Low

Risk of Bias (Newcastle-Ottawa Scale)

On the NOS, the included studies were generally of moderate-to-high methodological quality, with most scoring seven to nine of nine stars (Table 5). Strengths common to the cohort designs included representative population-based sampling, objective record-based ascertainment of exposure and outcome, and adequate follow-up. The most frequent limitations were in the comparability domain, reflecting incomplete adjustment for confounders such as healthcare-seeking behaviour and prior testing intensity, and in the adequacy of follow-up, given the relatively short observation windows in several studies.

**Table 5:** Risk-of-bias assessment using the Newcastle-Ottawa Scale

Study, Ref	Selection (4)	Comparability (2)	Outcome (3)	Total (9)	Overall quality
Chang [12]	4	1	3	8	High
Tesch [13]	4	2	2	8	High
Syed [14]	4	2	2	8	High
Lim [15]	4	2	2	8	High
Peng [16]	4	2	2	8	High
Hileman [17]	3	1	2	6	Moderate
Kim [18]	4	2	3	9	High
Heo [19]	4	2	3	9	High

Discussion

This systematic review of eight large, controlled cohort studies indicates that confirmed SARS-CoV-2 infection is associated with an increased risk of new-onset autoimmune and autoinflammatory disease across a remarkably broad clinical spectrum. The consistency of the direction of effect is the most robust finding: despite differences in geography, data source, comparator, and disease ascertainment, every included study reported elevated risk of one or more incident autoimmune conditions after COVID-19 [12-19]. This convergence across independent national health systems--the United States [12,17], Germany [13], the United Kingdom [14], South Korea [15,18,19], Japan [18], and Hong Kong [16]--makes it unlikely that the association is an artefact of any single healthcare context or coding practice. At the same time, the magnitude of the association varied by nearly an order of magnitude between studies, a heterogeneity that must temper any uniform interpretation and that warrants careful mechanistic and methodological discussion.

The epidemiological signal is biologically plausible. SARS-CoV-2 infection induces new-onset immunoglobulin G autoantibodies in a substantial proportion of hospitalised patients, with reactivities that develop de novo and correlate with the magnitude of the antiviral response [20]. The breadth of these autoantibody responses prompted the early conceptualisation of SARS-CoV-2 as an “autoimmune virus,” reflecting the strong association between infection and autoimmunity recognised from the first year of the pandemic [21]. Mechanistically, molecular mimicry between SARS-CoV-2 and human antigens offers a unifying explanation, and computational interactome analyses have identified shared epitopes and plausible autoimmune targets distributed across multiple organ systems [22]. Together, these mechanistic studies describe a credible pathway from acute infection to the breaking of self-tolerance, of which the population-level cohort findings synthesised here may represent the clinical expression. Importantly, these mechanisms are not mutually exclusive; molecular mimicry, bystander activation driven by intense innate inflammation, and the unmasking of latent autoreactivity by persistent viral antigen may act in concert, which could help to explain why the autoimmune conditions implicated across the included studies span such diverse organ systems rather than clustering within a single pathway.

The conditions most frequently implicated across the included studies align closely with the case-level literature. The marked elevations in rheumatoid arthritis, systemic lupus erythematosus, and connective tissue disease reported by Chang et al. [12], together with the vasculitic predominance in the analyses by Hileman et al. [17] and Peng et al. [16], echo an earlier systematic review of individually reported cases in which vasculitis and arthritis were the most common new-onset rheumatic manifestations after COVID-19, followed by inflammatory myopathies, lupus, and sarcoidosis [23]. The Korean connective tissue analyses extended this spectrum to alopecia, vitiligo, psoriasis, and inflammatory bowel disease, and demonstrated that risk scaled with acute severity [15,19]. This concordance between aggregated case descriptions and controlled cohort estimates strengthens confidence that the implicated conditions reflect a genuine biological signal rather than ascertainment alone.

The direction and approximate magnitude of the pooled association are corroborated by a recent systematic review and meta-analysis of population-based cohort studies encompassing approximately 97 million individuals, which quantified an increased relative risk of new-onset autoimmune disease following COVID-19, rating the certainty of evidence as moderate for outcomes with large effects but low overall owing to heterogeneity and the non-randomised designs [24]. That this independent quantitative synthesis reached conclusions consistent with the present narrative review, and with the composite estimates from the German claims-based [13] and United Kingdom primary-care [14] cohorts, lends external validity to our findings, while reinforcing the central caveat that pooled estimates conceal meaningful variation across populations and methods.

A particularly consistent and clinically salient theme is the relationship between acute infection severity and subsequent autoimmune risk. Among the included studies, the Korean connective tissue analyses [15,19] and the binational study [18] each reported a greater risk after more severe COVID-19. This exposure-response gradient was reproduced in a large United States RECOVER initiative study across three independent electronic health-record networks, in which patients with the most severe acute COVID-19 had the highest risk of new autoimmune disease, with inflammatory arthritis the most common adult diagnosis, and incident type 1 diabetes and haematological autoimmune disease most evident in children [24,25]. The reproducibility of the severity gradient across distinct national datasets and age groups is mechanistically coherent, given that more severe COVID-19 is characterised by a greater inflammatory and autoantibody burden [20], and it identifies severely affected patients as the subgroup warranting the closest post-infection surveillance.

Several findings nonetheless caution against overinterpretation. The binational study is instructive because it incorporated an influenza comparator in addition to uninfected controls and reproduced its results across two national datasets, yet it also demonstrated that the excess risk attenuated over the 12 months of follow-up [18]. This temporal attenuation raises the possibility that part of the apparent association reflects the earlier diagnosis of disease that would have emerged regardless of infection. Surveillance and detection bias is pervasive in this literature because individuals diagnosed with COVID-19 enter into more frequent medical contact, increasing the likelihood that an incipient autoimmune disease is detected and coded. The protective association between vaccination and several post-infection autoimmune outcomes reported in the Hong Kong cohort [16] is biologically encouraging and consistent with the hypothesis that attenuating infection severity reduces the downstream risk, although residual confounding by health behaviour cannot be excluded.

The heterogeneity in effect magnitude merits specific consideration. The substantially larger hazard ratios reported by Chang et al. [12] relative to the more modest estimates from Peng et al. [16] likely reflect differences in comparator construction, baseline testing intensity, follow-up duration, and the dominant circulating variant during each study window; the Hong Kong cohort, conducted largely in the Omicron era, sampled a population with milder average disease that may have diluted the apparent association. Such contextual differences caution against the naive pooling of estimates and explain the value of comparator choice, since studies benchmarked against another respiratory infection [18] provide more interpretable estimates of SARS-CoV-2-specific risk than those comparing against uninfected populations alone.

A further dimension that deserves emphasis is the role of demographic modifiers and the distinction between relative and absolute risk. Several analyses reported that the excess autoimmune risk after COVID-19 differed by age, sex, and race, with a female preponderance for inflammatory arthritis that mirrors the well-recognised sex distribution of autoimmune disease in general [12,15]. Yet even where adjusted hazard ratios were two- to three-fold elevated, the absolute incidence of any individual autoimmune condition over the follow-up periods studied remained low, such that the population-attributable burden, non-trivial given the sheer number of people infected worldwide, does not translate into a high individual probability of disease for most patients. Communicating this distinction between relative and absolute risk is critical to avoid undue alarm while still justifying appropriate post-infection vigilance and the prioritisation of severe cases for follow-up.

Taken together, the evidence supports a clinically meaningful but provisional conclusion: SARS-CoV-2 infection is associated with an increased risk of incident autoimmune disease, the association is strongest for connective tissue, vasculitic, and antibody-mediated disorders, and it is modulated by infection severity, time since infection, and possibly vaccination. The implications for practice are that clinicians should remain alert to new autoimmune presentations in the post-acute period after COVID-19, particularly in patients who experienced severe acute illness, while recognising that the absolute risk for any individual condition remains low. The implications for research are that future studies should prioritise active outcome ascertainment with serological and clinical confirmation, incorporate infection comparators to control for surveillance effects, extend follow-up to distinguish transient from durable risk, and stratify by variant and vaccination status. Only with such designs can the field move from demonstrating association toward establishing causation and quantifying attributable risk with confidence. In parallel, the identification of serological or transcriptomic biomarkers capable of flagging individuals at elevated risk would allow post-infection surveillance to be targeted rather than applied indiscriminately, improving both its diagnostic yield and its cost-effectiveness.

Limitations

This review has several limitations that should be acknowledged. First, the review protocol was not prospectively registered (e.g., in PROSPERO), which may reduce methodological transparency and introduce the possibility of selective reporting, although the review was conducted according to a predefined methodology and followed PRISMA guidelines. In addition, all included studies were retrospective observational cohort studies, with no prospective studies identified. Consequently, the findings are inherently susceptible to residual confounding, surveillance bias, misclassification of both exposure and outcomes, and reverse causation, despite statistical adjustment in several studies. These methodological limitations reduce the certainty with which causal inferences can be drawn regarding the association between COVID-19 infection and subsequent autoimmune disease.

Furthermore, heterogeneity in exposure definitions, comparator groups, outcome case definitions, follow-up duration, healthcare settings, and circulating SARS-CoV-2 variants precluded a valid quantitative meta-analysis, and the certainty of evidence assessed using GRADE was correspondingly low to moderate. The evidence base was also geographically skewed toward East Asian and high-income Western populations, limiting the generalisability of the findings to other regions. In addition, several studies had relatively short follow-up periods, which may not have been sufficient to capture autoimmune diseases with a delayed or indolent onset. Finally, this review was restricted to peer-reviewed, English-language, controlled studies. Although this approach was intended to enhance methodological rigour, the exclusion of non-English publications, preprints, and case reports may have resulted in the omission of relevant evidence, particularly for rare autoimmune manifestations. Moreover, publication bias could not be formally assessed because no quantitative meta-analysis was performed, and the number and methodological heterogeneity of the included studies precluded meaningful use of funnel plots or statistical tests such as Egger's regression. Therefore, the possibility that studies reporting positive associations between COVID-19 and autoimmune disease were more likely to be published than those reporting null findings cannot be excluded.

## Conclusions

Synthesising eight large controlled cohort studies, this systematic review found that confirmed COVID-19 infection is consistently associated with an increased risk of new-onset autoimmune and autoinflammatory disease, most reproducibly affecting connective tissue, vasculitic, and antibody-mediated disorders, with risk modified by acute disease severity, time since infection, and vaccination. These findings, biologically supported by the autoantibody and molecular-mimicry mechanisms documented in mechanistic studies, justify clinical vigilance for autoimmune sequelae after COVID-19 while underscoring that the present evidence remains observational and of limited certainty. Rigorously designed prospective studies with infection comparators, validated outcomes, and extended follow-up are needed to confirm causality and to define the long-term autoimmune burden of the COVID-19 pandemic.

## Appendices

**Table 6:** Search strings used for the searched databases

Database	Search String
MEDLINE (PubMed)	("COVID-19"[Mesh] OR "COVID-19"[tiab] OR "SARS-CoV-2"[tiab] OR "2019-nCoV"[tiab] OR "coronavirus disease 2019"[tiab]) AND ("Autoimmune Diseases"[Mesh] OR autoimmune[tiab] OR "autoimmune manifestation*"[tiab] OR "autoimmune disorder*"[tiab] OR "autoimmunity"[tiab] OR "post-infectious autoimmunity"[tiab] OR "immune-mediated disease*"[tiab])
Embase	('covid-19'/exp OR 'covid-19':ti,ab OR 'sars-cov-2':ti,ab OR '2019 ncov':ti,ab OR 'coronavirus disease 2019':ti,ab) AND ('autoimmune disease'/exp OR autoimmune:ti,ab OR 'autoimmune manifestation*':ti,ab OR 'autoimmune disorder*':ti,ab OR autoimmunity:ti,ab OR 'immune mediated disease*':ti,ab OR 'post infectious autoimmunity':ti,ab)
Web of Science	TS=(("COVID-19" OR "SARS-CoV-2" OR "2019-nCoV" OR "coronavirus disease 2019") AND ("autoimmune" OR "autoimmune disease*" OR "autoimmune disorder*" OR "autoimmune manifestation*" OR "autoimmunity" OR "immune-mediated disease*" OR "post-infectious autoimmunity"))
Cochrane Library	("COVID-19" OR "SARS-CoV-2" OR "coronavirus disease 2019"):ti,ab,kw AND ("autoimmune" OR "autoimmune disease*" OR "autoimmune disorder*" OR "autoimmune manifestation*" OR "autoimmunity" OR "immune-mediated disease*"):ti,ab,kw
